# Estimating capacity requirements for substance use treatment systems: a population-based approach

**DOI:** 10.1186/1940-0640-10-S1-A58

**Published:** 2015-02-20

**Authors:** Brian Rush, Joel Tremblay, Chantal Fougere

**Affiliations:** 1 Centre for Addiction and Mental Health, Toronto, Ontario, Canada, M5S 2S1; 2 Université du Québec à Trois-Rivières, Trois-Rivières, Québec, Canada, G9A 5H7

## Background

Treatment system planning and resource allocation is hampered by the lack of systems-level data and planning frameworks. We developed and pilot-tested a needs-based planning model for substance use services and supports that aligns with the estimated needs of the population of local health regions, takes a broad systems approach beyond the specialized sector, and yields estimates of required treatment capacities for service categories along the continuum of care.

## Method

Using national population survey data, we estimated, for 94 regional planning areas in Canada, the number of people in need of substance use treatment within a given year, based on five ‘tiers’ of problem severity. We then estimated the probable help-seeking population for each level of severity, based on a synthesis of the literature. Working with a national expert consensus panel, we estimated the optimal trajectory of clients across several defined categories of treatment services organized by level of care. Integrating steps 1–3 yielded the number of people to plan for in each service setting. We piloted the model in nine Canadian jurisdictions, conducting gap analyses that compared the projected and actual service utilization, and supplemented by stakeholder feedback and local indicators of need, such as wait lists and referral data.

## Results

The model development process and gap analyses at the nine pilot sites yielded important results for local planners, but with national implications. Results indicated that the capacity of moderate-intensity services is adequate in many regions, but that larger gaps exist in low-threshold services (e.g., home-based/mobile withdrawal management) and high-intensity services (e.g., medical inpatient services for high-complexity cases. These results and their implications were validated by stakeholders in the pilot sites.

## Conclusions

The needs-based planning model appears to have value in identifying local gaps in services, but regional context must be taken into account when applying the model to local jurisdictions. The piloting process highlighted a national need for systematic screening and brief intervention processes in the nonspecialized sector to improve early identification and referral of clients. We anticipate that the model will serve as a valuable tool for system planners to use in discussions and decisions about funding and resource allocation. Next steps include model adjustments using more precise regional data, developing a separate component for opiate substitution, a youth version, and incorporating the model into a larger needs assessment process. Comparable work is underway in other countries (e.g., Australia, Brazil, UK), providing opportunities for international knowledge exchange.

